# Quinine in the Treatment of Whooping Cough

**Published:** 1883-09

**Authors:** 


### QUININE IN THE TREATMENT OF WHOOPING COUGH.

Dr. W. Thornton Parker, Surgeon United States Army, writes most favorably of his experience of the use of quinine in the treatment of whooping cough. He gives teaspoonful doses of a solution of quinine of four, six, eight, or even ten grains to the ounce, every two hours, and finds that it gives *almost immediate relief* to the little sufferers. This method of treatment was brought from Germany to New York by Dr. Dawson some five years ago, and used by him with great success in the wards of the Hospital for Children in New York City, then under his care. Dr. Parker questions Dr. Dolan's assertion (in his Fothergillian essay on Whooping Cough) that the course of the disease could not be controlled by treatment. His experience leads him to believe that in quinine, in the dosage recommended, we have a remedy capable of exercising control, and even of cutting short the disease.

---

NEVER put pickles in a jar that has had lard in it.

BEESWAX and salt will make rusty flat-irons as smooth as glass.

FISH may be scaled much more easily if dipped for an instant in boiling water.

IT will rest you wonderfully to change your seat in the room occasionally if you have a long day's sewing to do.

TOUGH meat may be made as tender as any by the addition of a little vinegar to the water when it is put on to boil.

IT soothes and cools a feverish patient to bathe him with warm water in which a little salaratus has been dissolved.

THE ground around dwelling houses should be paved, flagged asphalted, covered with concrete, or be graveled.

A BED of concrete over the site of cottages will vastly modify an otherwise objectionable position; but, indeed, a bed of concrete should be used in all cases.

NURSERIES and children's rooms should be permanently ventilated. Dormitories for children should have ample ventilation; clothe the children warmly, cover the beds warmly, prevent direct draughts, and the cool air will not injure.

A LONG RIDE.—Prof. Young, of Princeton College, thus illustrates the distance from the earth to the sun: "Take a railroad from the earth to the sun, with a train running forty miles an hour without stops, and it would take about two hundred and sixty-five years and a little over to make the journey." He estimated the fare, at a cent a mile, to be nine hundred and thirty thousand dollars.

